# Imitators of chronic pancreatitis: diffuse neuroendocrine tumour of the pancreas

**DOI:** 10.1259/bjrcr.20170015

**Published:** 2017-07-29

**Authors:** Haresh Naringrekar, Ashley Vogel, Anthony Prestipino, Haroon Shahid

**Affiliations:** ^1^Department of Radiology, Einstein Radiology, Einstein Medical Center, Philadelphia, PA, USA; ^2^Department of Pathology, Thomas Jefferson University Hospital, Philadelphia, PA, USA; ^3^Department of Gastroenterology, Thomas Jefferson University Hospital, Philadelphia, PA, USA

## Abstract

We report a rare case of diffuse replacement of the pancreas with neuroendocrine tumour mimicking chronic pancreatitis. A 55-year-old female with no significant past medical history initially presented with abdominal pain in 2006. A CT of the abdomen and pelvis was performed, revealing diffuse pancreatic parenchymal calcifications with mild pancreatic ductal dilatation and no discrete mass. She was diagnosed with chronic pancreatitis and followed clinically until 2015, where she presented with recurrent abdominal pain. A repeat CT and MRI of the abdomen were performed which revealed new hypoenhancing masses within the pancreas, particularly in the pancreatic tail. There was a persistent background of pancreatic parenchymal calcifications. The possibility of pancreatic neuroendocrine tumour was raised, and an indium-111 Octreotide scan was recommended. Diffuse intense uptake was identified throughout the pancreas on the indium-111 imaging. Given the concern for neuroendocrine tumour, a total pancreatectomy was performed, with histopathology revealing replacement of the pancreas with coalescing well-circumscribed nodules. Many of the nodules had numerous calcifications and localized amyloid deposition. Immunohistochemical stains of the neoplastic cells were strong for neuroendocrine markers chromogranin A and synaptophysin. Overall the findings were consistent with numerous neuroendocrine tumours of the pancreas, Grade II, as per the 2010 WHO criteria for neuroendocrine tumours of the pancreas. Neuroendocrine tumours of the pancreas are lesions that arise from the islet cells, with an approximate incidence of five cases per million people per year. Only one other case report has been documented in the literature by Singh et al demonstrating diffuse pancreatic neuroendocrine tumour replacing the entire pancreas. As diffuse pancreatic neuroendocrine tumour can look similar on imaging to chronic pancreatitis or other infiltrative processes, we wanted to present this case and some of the more specific imaging findings in distinguishing these entities.

## Case report

**Figure 1.**
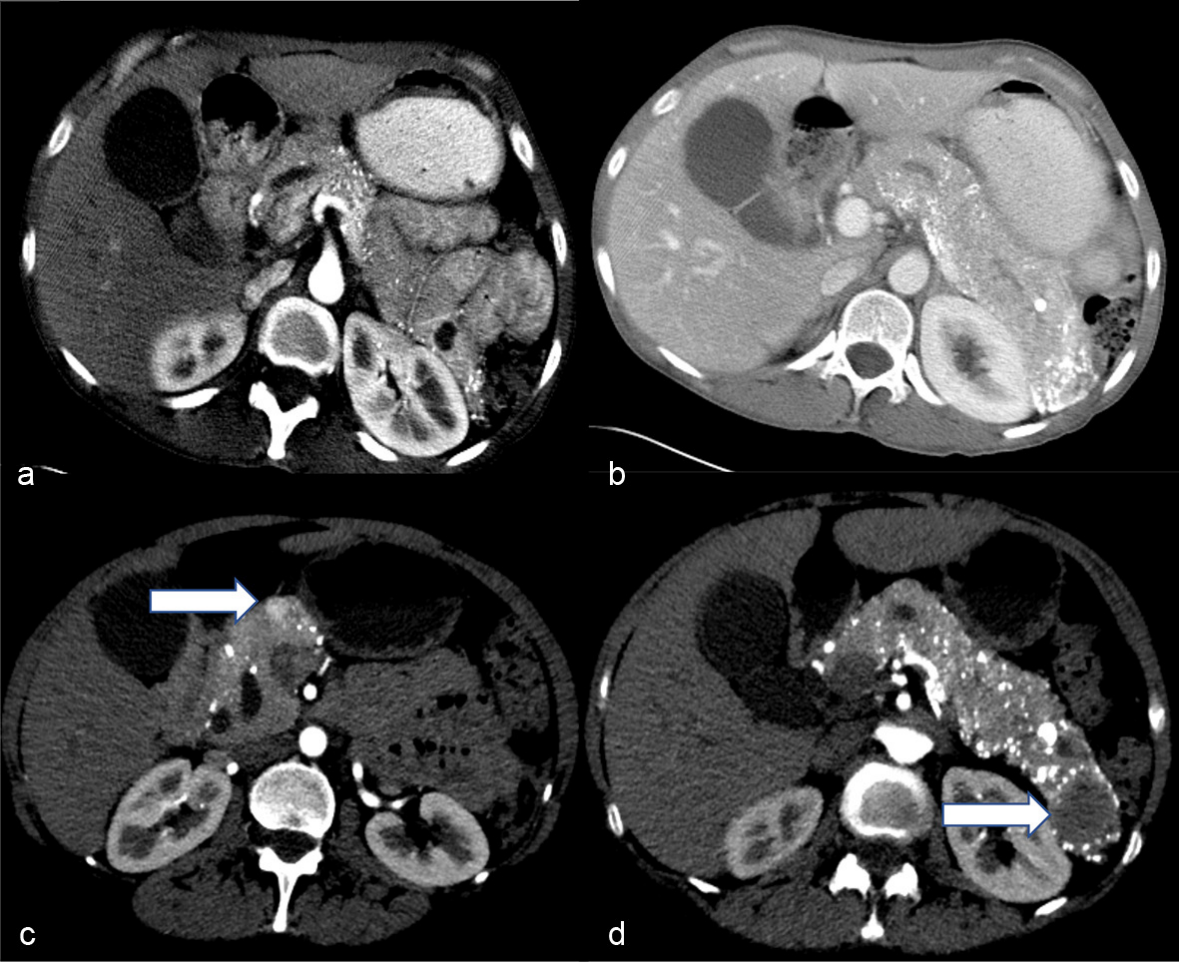
(a-d) Axial arterial (a) and portal venous contrasts enhanced. (b) CT images through the pancreas obtained in 2006 using a pancreatic mass protocol show diffuse pancreatic parenchymal calcifications with mild pancreatic ductal dilatation and no discrete mass. Contrast-enhanced CT images through the pancreatic head (c) and body (d) obtained in 2015 reveal persistent parenchymal calcifications and mild pancreatic duct dilatation. Note the lack of parenchymal atrophy in 2015. Development of a new arterially enhancing mass in the pancreatic neck (arrow on c) and hypodense mass in the pancreatic tail (arrow in d) is identified.

**Figure 2.**
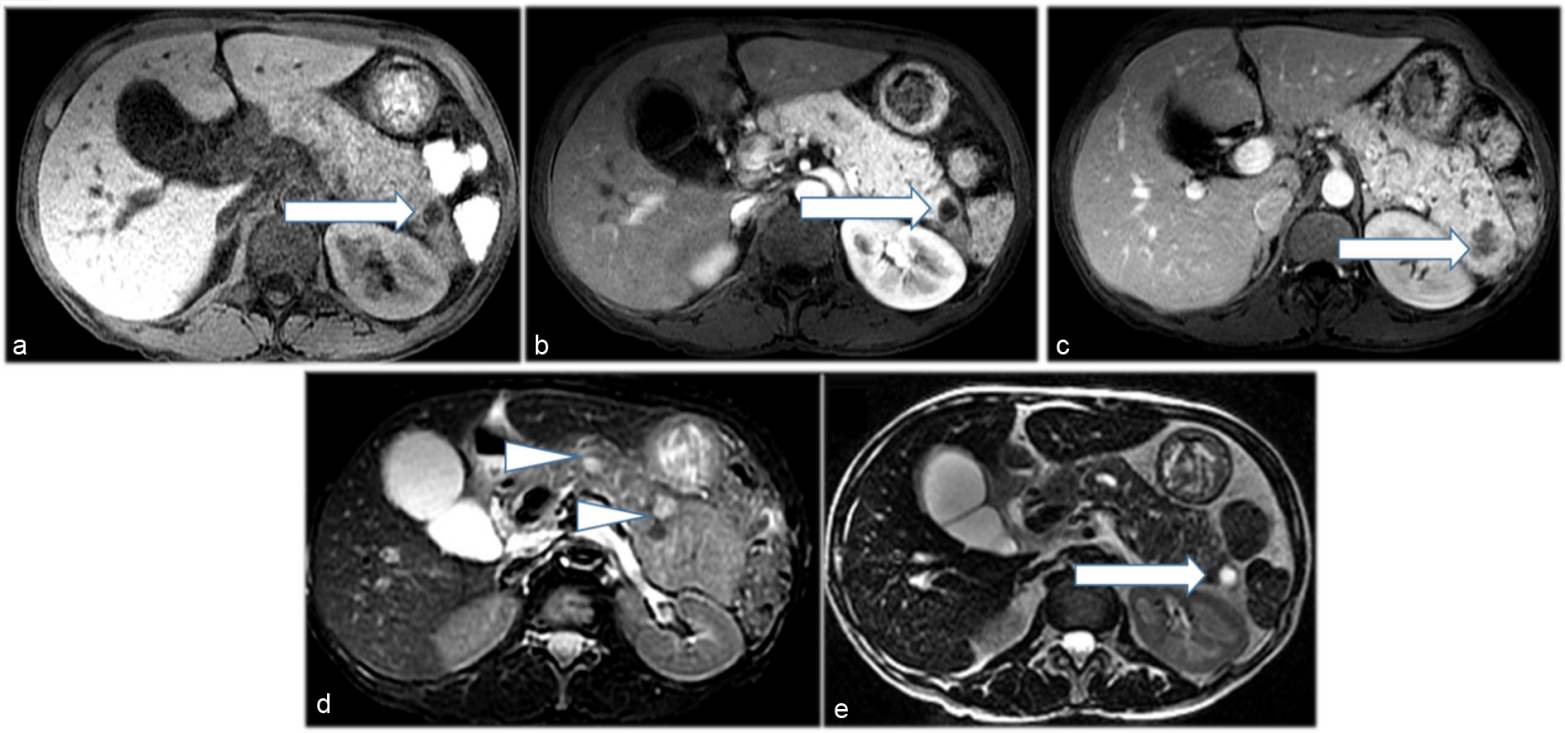
( a-e) Pre-contrast *T*_1_ weighted image with fat saturation (a) shows normal intrinsic *T*_1_ signal within the pancreas, with a *T*_1_ hypointense mass in the pancreatic tail (arrow). (b) Post-contrast arterial phase *T*_1_ weighted image reveals brisk enhancement of the pancreas with a relative hypointense mass in the pancreatic tail (arrow). Post-contrast *T*_1_ weighted image with fat saturation in portal venous phase (c) demonstrates no delayed enhancement of the pancreas, with increased irregular enhancement of the pancreatic tail mass (arrow). *T*_2_ weighted sequences with fat saturation (d) and without fat saturation (e) show the pancreatic tail mass to be *T*_2_ hyperintense (arrows). Additional *T*_2_ hyperintense masses are noted in the pancreatic neck and distal body (arrowheads in d).

**Figure 3.**
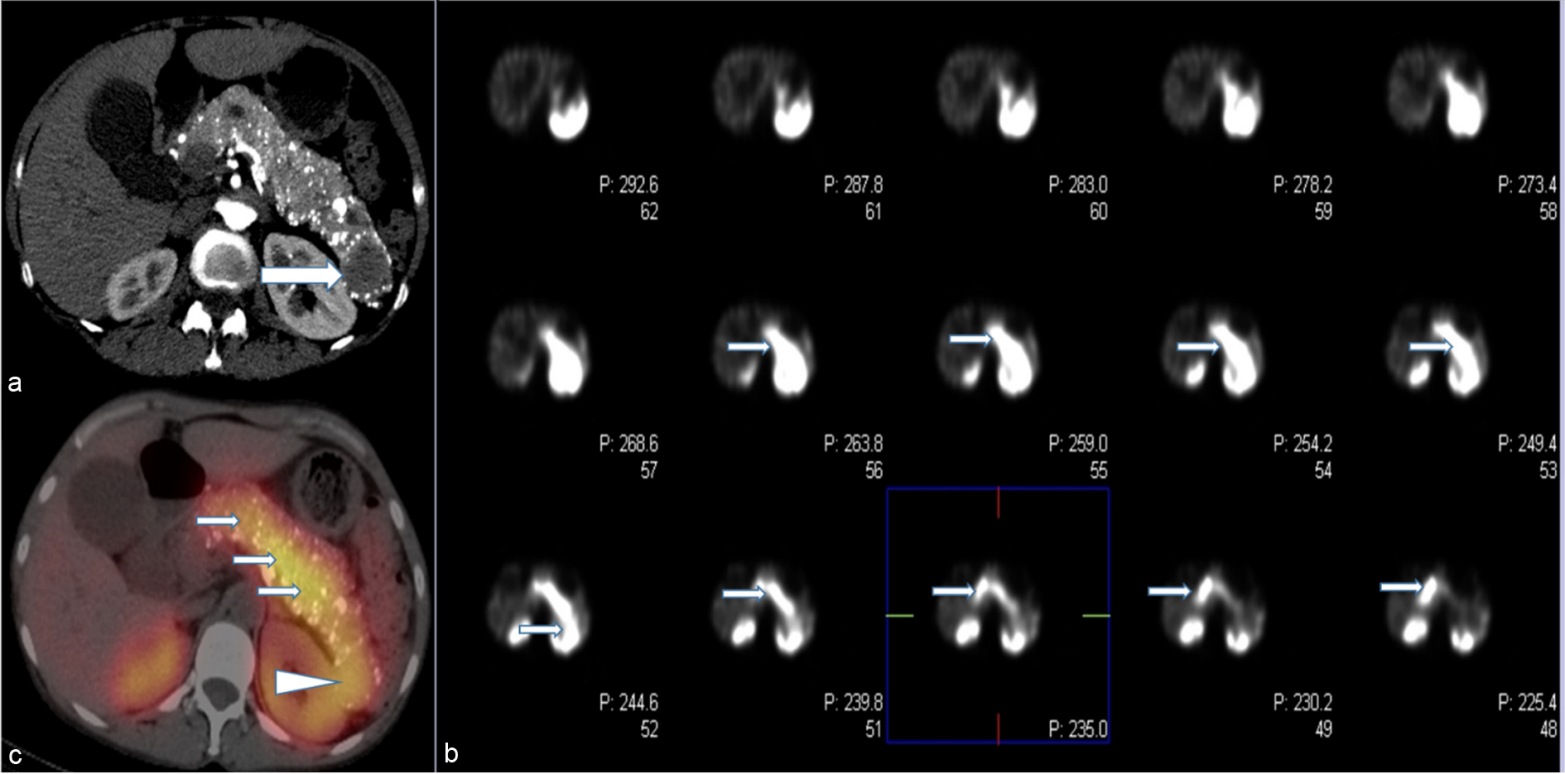
(a-c) Contrast-enhanced arterial phased CT image through the pancreas obtained in 2015 (a) show diffuse parenchymal calcifications with a more discrete mass in the pancreatic tail (arrow). SPECT images of the abdomen from an indium-111 Octreotide scan through the pancreas (b) show diffuse uptake of radiotracer throughout the entire pancreas (arrows). Fused SPECT-CT images from the same study (c) confirm diffuse uptake in the pancreas (arrow), including the pancreatic tail mass (arrowhead).

**Figure 4.**
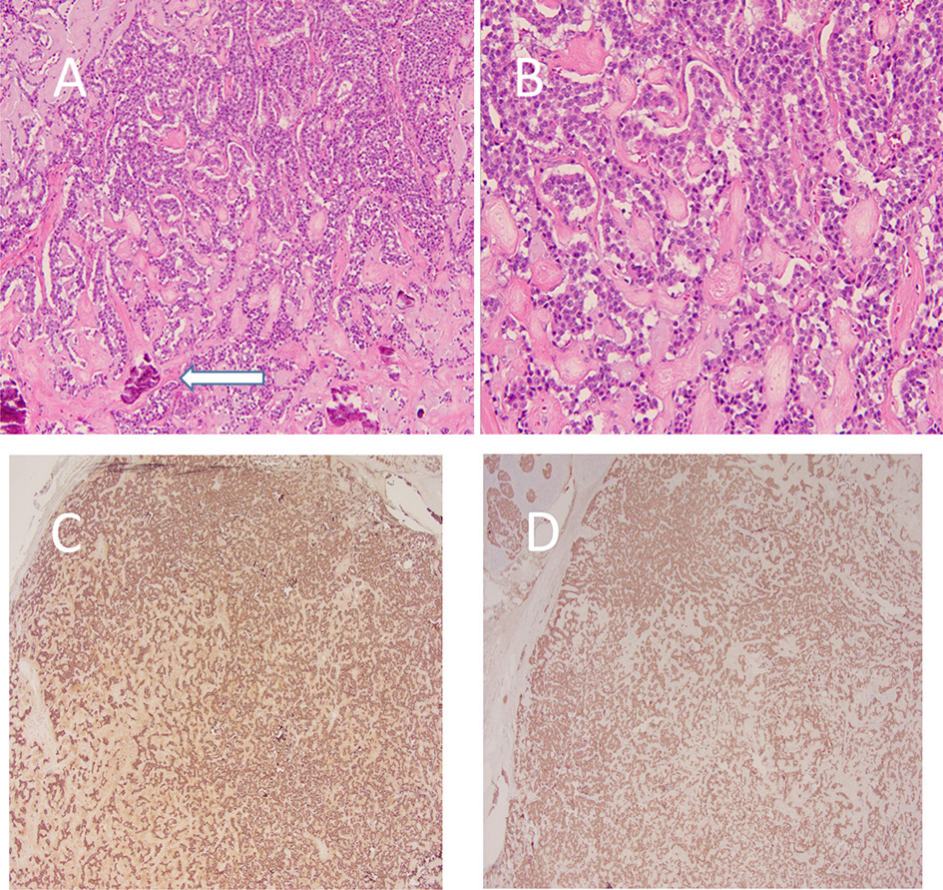
(a) 10x H&E. The nodules were comprised of trabecular cords and insular nests of fairly uniform epitheliod cells with surrounding deposition of amyloid matrix (confirmed by Congo Red special stain) and multiple calcifications (white arrow). 20x H&E. (b) Higher power view of the cords of epithelial cells with surrounding amyloid matrix. No significant pleomorphism or mitotic activity was identified on multiple representative sections. 2x IHC. (c) Immunohistochemical stain for neuroendocrine marker chromogranin A shows diffuse cytoplasmic staining of the neoplastic cells. 2x IHC. (d) Immunohistochemical stain for neuroendocrine marker synaptophysin shows diffuse cytoplasmic staining of the neoplastic cells.

A 55-year-old female with no significant past medical history initially presented in 2006 with vague abdominal pain. Her past social history was negative for alcoholism or smoking. Work up of the patient including physical examination and laboratory indices were all normal. A multi-detector CT of the abdomen and pelvis utilizing a pancreatic mass protocol (arterial, portal venous and delayed phases) was performed which revealed a diffusely enlarged pancreas with extensive parenchymal calcifications (Figure 1). No discrete mass was identified in the pancreas. Minimal pancreatic ductal dilatation was noted on the CT examination. The patient was diagnosed with chronic pancreatitis and followed clinically for several years. In 2015, the patient presented with recurrent abdominal pain. A CT of the abdomen and pelvis with pancreatic mass protocol was repeated (Figure 1), which revealed new hypoattenuating masses in the pancreatic neck and tail. Some of the smaller tumours were hypervascular on the arterial phase imaging. There was redemonstration of extensive parenchymal calcifications. No pancreatic atrophy was identified. The pancreatic duct remained minimally dilated. Given the new masses many of which were arterially enhancing, the possibility of neuroendocrine tumour was raised. A MRI of the abdomen with pancreatic mass protocol (*T*_2_ with fat saturation, MRCP, in and out of phase *T*_1_ and unenhanced *T*_1_/arterial/portal/ 5 min delayed post-contrast *T*_1_) was performed (Figure 2). The MRI confirmed multiple well-circumscribed masses throughout the pancreas, many of which had increased *T*_2_ signal with cystic change. The pancreatic duct was at most mildly prominent, and no lesions were identified outside of the pancreas. The largest pancreatic mass in the tail measured approximately 3.5 cm, with peripheral enhancement and central hypointense signal (Figure 2). An endoscopic ultrasound was performed, which confirmed a hypoechoic mass in the tail of the pancreas, two isoechoic masses in the head of the pancreas, and diffuse parenchymal calcifications suggestive of chronic pancreatitis. Fine needle aspiration of the pancreatic masses in the head was performed, with cytology returning as concerning for neuroendocrine tumour. Given the suspicion for pancreatic neuroendocrine tumour, an indium-111 Octreotide scan was requested for further characterization. Fused SPECT-CT imaging was also performed for improved uptake localization (Figure 3). The indium-111 scan revealed diffuse intense uptake of radiotracer throughout the entire pancreas. No extrapancreatic foci of uptake was identified. On the grounds of the clinical and imaging findings, it was decided the best course of action would be to perform a pylorus-preserving pancreaticoduodenectomy with total resection of the pancreas, splenectomy and cholecystectomy. Sectioning of the pancreas revealed numerous well-circumscribed, solid and tumoural masses ranging from minute up to the largest grossly identified lesion measuring 3.5 cm in diameter (Figure 4). Many of the nodules were coalescing with only a scant amount of intervening normal pancreatic parenchyma present. The cut surfaces of the nodular masses were solid and showed a variegated pink to orange-red colour. No gross areas of necrosis were identified. Numerous representative histologic sections of the nodular masses were examined. The nodules were comprised of numerous insular nests and trabecular cords of fairly uniform epithelioid neoplastic cells with oval nuclei and speckled chromatin. Many of the nodules showed numerous calcifications and localized amyloid deposition (Figure 4a,b). Immunohistochemical stains were performed and the neoplastic cells marked strongly for the neuroendocrine markers chromogranin A and synaptophysin (Figure 4c,d). Multiple immunostains for pancreatic peptides were performed. The neoplastic cells were positive for pancreatic polypeptide and negative for insulin, glucagon and somatostatin. Only a rare mitotic figure was identified but the Ki-67 mitotic index marker was calculated at 5% as measured by the Aperio image analysis system. The findings were consistent with numerous neuroendocrine tumours of the pancreas, Grade II, as per the 2010 WHO criteria for neuroendocrine tumours of the pancreas. The neuroendocrine neoplastic nodules were all confined within the pancreatic parenchyma and all pancreatic resection margins were free of neoplasia. All regional lymph nodes sampled were negative for metastatic disease.

## Discussion

Pancreatic neuroendocrine tumours are rare tumours arising from pancreatic islet-cells, with an incidence of approximately five cases per million per year.^1^ These tumours represent about 1.5% of all gastrointestinal and pancreatic neoplasms.^2–4^ The majority of pancreatic neuroendocrine tumours have no known risk factors. Increased incidence has been seen with women, African Americans, Hispanics and Asians. Associations with conditions such as hypergastrinemia, pre-existing diabetes mellitus and ulcerative colitis are also noted; however, definite origins are not known currently.^5–8^ Systemic syndromic associations with pancreatic neuroendocrine tumours include Multiple Endocrine Neoplasia Type 1 (MEN 1 or Wermer’s syndrome), neurofibromatosis 1 (NF-1 or von Recklinghausen disease), tuberous sclerosis and Von Hippel Lindau disease.

Clinical presentation depends on whether the tumour secretes hormones or not. Tumours that secrete hormones tend to present earlier due to the earlier symptomatic presentation, with common syndromic tumours including insulinomas, gastrinomas, glucagonomas and VIPomas. These tumours tend to be small, discrete arterially enhancing masses on CT and MRI. Non-hormone producing tumours tend to present clinically as abdominal pain as in our patient due to mass effect. On imaging these tend to present as larger masses with calcifications, cysts, necrosis and vascular invasion.^9^ Our case presented a challenging imaging and clinical dilemma as the patient had non-functioning tumour replacing the entire pancreas with diffuse calcification, mimicking chronic pancreatitis or amyloidosis. Only one other case has been documented to the best of our knowledge showing diffuse replacement of the pancreas with neuroendocrine tumour.^1^ Clinically, the patient presented only with recurrent abdominal pain; she had no history of diabetes, diarrhea or malnourishment, symptoms often seen in patients with chronic pancreatitis.^10^ Additionally, her social history was negative for any significant consumption of alcohol, one of the more significant risk factors for chronic pancreatitis.^10^ The interval development of masses on a background of apparent chronic pancreatitis was also highly unusual.

There are several distinguishing features between etiologies such as chronic pancreatitis and our case of diffuse pancreatic neuroendocrine tumour. Late findings of chronic pancreatitis present on imaging with diffuse calcifications particularly in the pancreatic duct with *atrophy *of the pancreas. The temporal imaging of our case showed no atrophy of the pancreas over a 10-year period, with the pancreas remaining diffusely enlarged, pointing to another diagnosis. The calcifications were all parenchymal, with no intraductal stones. Additionally, late findings of chronic pancreatitis show diffuse moderate to severe dilatation of the pancreatic duct with irregular beading and dilatation of the side branches, another finding absent in our case. The pancreas in our patient had mild pancreatic ductal dilatation which was stable over several years. Chronic pancreatitis diminishes proteinaceous content in the parenchyma, which manifests as diffusely low *T*_1_ signal on MRI.^11^ The enhancement patterns show decreased arterial enhancement, with relative increased delayed enhancement on later phase post-contrast sequences.^12–16^ With the exception of the more discrete tumours found in our case, the *T*_1_ signal and enhancement pattern of the pancreas was relatively normal. Finally, the formation of discrete enhancing masses with increased *T*_2_ signal within the pancreas should point to another diagnosis. Nuclear medicine helped play a definitive roll in pointing towards the diagnosis neuroendocrine tumour. Given the hormonal activity of many neuroendocrine tumours of the pancreas, there is often an expression of somatostatin receptors. Scintography takes advantage of this by labeling Octreotide, a somatostatin analogue, with a radiotracer, most commonly indium-111. Tumours that express somatostatin receptors will uptake the radiotracer, and this can be localized with SPECT and SPECT/CT fused imaging. A relatively newer method for imaging neuroendocrine tumours takes advantage of PET/CT and is known as Ga 68-DOTA PET imaging. This method has several advantages over traditional imaging, namely improved spatial resolution/tumour localization, cheaper to use (no need for cyclotron), less time to image and ability to measure the level of uptake.^17^ Unfortunately, this newer technique was unavailable at our institution; traditional indium-111 Octreotide scan was performed which demonstrated diffuse uptake of radiotracer in the pancreas particularly in the *T*_2_ hyperintense masses, consistent with the favoured neuroendocrine tumour rather than chronic pancreatitis.^9^

## Conclusions

We report a rare case of diffuse replacement of the pancreas with neuroendocrine tumour mimicking chronic pancreatitis. In this scenario, we present the imaging findings that point towards a diagnosis of neuroendocrine tumour rather than the patient’s presumed diagnosis of chronic pancreatitis.

## Learning points

1. Diffuse neuroendocrine tumour is a rare mimic of chronic pancreatitis.
2. Neuroendocrine tumour can be differentiated by other causes of diffuse pancreatic calcification by the lack of pancreatic atrophy and predominant parenchymal with lack of ductal calcifications.
3. Nonfunctioning neuroendocrine tumours can reach a much larger size before being discovered due to the lack of physiologic symptoms from hormones.
4. Indium-111 Octreotide imaging can help provide non-invasive imaging method for suggesting neuroendocrine tumour of the pancreas, particularly in the setting of diffuse replacement with tumour. Nuclear medicine techniques like indium-111 Octreotide imaging or the newer Ga 68-DOTA PET imaging can help provide non-invasive imaging method for suggesting neuroendocrine tumour of the pancreas, particularly in the setting of diffuse replacement of pancreas with tumour.

## Consent

Written informed consent for the case to be published (including images, case history and data) was obtained from the patient(s) for publication of this case report, including accompanying images.
